# Supervised exercise training as an adjunctive therapy for venous leg ulcers: study protocol for a randomised controlled trial

**DOI:** 10.1186/s13063-015-0963-z

**Published:** 2015-10-06

**Authors:** Garry A. Tew, Jonathan Michaels, Helen Crank, Geoff Middleton, Anil Gumber, Markos Klonizakis

**Affiliations:** York Trials Unit, Department of Health Sciences, University of York, Heslington, York, YO10 5DD UK; Health Economics and Decision Science, ScHARR, University of Sheffield, Regent Court, 30 Regent Street, Sheffield, S1 4DA UK; Centre for Sport and Exercise Science, Collegiate Hall, Sheffield Hallam University, Collegiate Crescent, Sheffield, S10 2BP UK; School of Sport and Exercise Science, College of Social Science, University of Lincoln, Brayford Campus, Lincoln, LN6 7TS UK; Centre for Health and Social Care Research, Sheffield Hallam University, Montgomery House, Collegiate Crescent, Sheffield, S10 2BP UK

**Keywords:** Venous ulcers, Venous insufficiency, Physical therapy, Exercise therapy, Rehabilitation, Randomized controlled trial, Feasibility studies

## Abstract

**Background:**

Venous leg ulcers are common, chronic wounds that are painful and reduce quality of life. Compression therapy is known to assist in the healing of venous leg ulceration. Supervised exercise training that targets an improvement in calf muscle pump function might be a useful adjunctive therapy for enhancing ulcer healing and other aspects of physical and mental health. However, the evidence of exercise for individuals with venous ulcers is sparse. Here, we describe the protocol for a study that aims to assess the feasibility of undertaking a randomised controlled trial of a supervised exercise programme in people who are receiving compression for venous ulceration.

**Methods/Design:**

This is a randomised, controlled, assessor-blinded, two-centre, feasibility trial with two parallel groups. Eighty adults who are receiving lower-limb compression for a venous leg ulcer will be randomly assigned to receive usual care (compression only) or usual care plus a 12-week supervised exercise programme. Participants in the exercise group will be invited to undertake three, 60-minute sessions of supervised exercise each week, and each session will involve a combination of treadmill walking, upright cycling and strength and flexibility exercises for the lower limbs. Participants will be assessed before randomisation and 3, 6 and 12 months after randomisation. Primary outcomes include rates of recruitment, retention and adherence. Secondary outcomes include time to ulcer healing, proportion of participants healed, percentage and absolute change in ulcer size, health-related quality of life (EQ-5D-5L and VEINES-QOL/Sym), lower-limb cutaneous microvascular function (laser Doppler flowmetry coupled with iontophoresis) and physical fitness (30-second sit-to-stand test, chair sit and reach test, 6-minute walk test and ankle range of motion). The costs associated with the exercise programme and health-care utilisation will be calculated. We will also complete interviews with a sub-sample of participants to explore their experiences of having a venous ulcer and the acceptability of the exercise intervention and study procedures.

**Discussion:**

Data from this study will be used to refine the supervised exercise programme, investigate the acceptability of the intervention and study design and determine the most appropriate outcome measures, thereby providing estimates of the factors needed to design an adequately powered trial across several centres.

**Trial registration:**

Current Controlled Trials, ISRCTN10205425 (May 2014) - http://www.controlled-trials.com/ISRCTN10205425

**Electronic supplementary material:**

The online version of this article (doi:10.1186/s13063-015-0963-z) contains supplementary material, which is available to authorized users.

## Background

Venous ulceration is the most common type of leg ulceration. The occurrence of venous leg ulcers increases with age, and the annual UK prevalence in those over 65 years is estimated at 1.7 % [1]. Venous ulcers arise from venous valve incompetence and calf muscle pump insufficiency which leads to venous stasis and hypertension. This results in microcirculatory changes and localised tissue ischaemia [2, 3]. The natural history of the disease is of a continuous cycle of healing and breakdown over decades, and chronic venous leg ulcers are associated with considerable morbidity and impaired quality of life [4]. Treatment of this major health problem results in a considerable cost to the National Health Service (NHS). The cost of treating one ulcer was estimated to be between £1,493 and £1,795 per year on the basis of 2010–2011 prices and in the context of a trial conducted within the UK NHS [5].

A first-line treatment for venous ulcers is compression therapy, the objective of which is to provide graded external compression to the leg and oppose the hydrostatic forces of venous hypertension [6]. Cochrane reviews have concluded that compression therapy is effective for promoting ulcer healing [7] and reducing the risk of ulcer recurrence [8]. O’Meara and colleagues [7] also concluded that multi-component systems delivering high compression (defined as 40 mm Hg of compression at the ankle) were the most effective treatment for venous ulcers. A recent trial demonstrated that two-layer compression hosiery is equally effective as four-layer bandaging for the healing of venous ulcers [5].

Lifestyle factors, including nutrition, exercise and smoking, are also mentioned in guidelines on the management of venous ulceration and chronic venous insufficiency (CVI) [9]; however, these factors receive relatively little emphasis. Exercise training that targets an improvement in calf muscle pump function might be a particularly useful adjunct therapy for enhancing ulcer healing and other aspects of physical and mental health. Exercise training is routinely prescribed for other forms of cardiovascular disease such as peripheral arterial disease [10] and coronary artery disease [11]. In patients with venous leg ulcers, supervised calf muscle exercise has been shown to increase calf muscle pump function and improve lower-limb haemodynamics [12, 13], and supervised treadmill walking exercise has been shown to improve lower-limb skin microvascular function in individuals with mild CVI [14]. However, a recent systematic review concluded that further research is required to determine whether exercise training has a beneficial effect on ulcer healing and health-related quality of life [15].

In light of this background evidence, we hypothesised that a specially designed supervised exercise programme delivered in conjunction with compression therapy would be a more effective means of promoting venous ulcer healing than compression therapy alone. Before embarking on an adequately powered randomised trial to test this hypothesis, we have planned a preliminary study that will address several areas of uncertainty. For example, leg pain and the perception that exercise might be harmful or too difficult to perform have been identified as barriers to exercise in people with venous ulcers [16], and this raises questions about how easy it will be to recruit and retain participants in a trial of a supervised exercise programme. The specific aims of the present feasibility study are the following:

1. Estimate the rates of recruitment and retention for a future definitive trial of a 12-week supervised exercise programme
2. Estimate the rates of attendance to and compliance with the supervised exercise programme
3. Estimate the outcome completion rate (provision of data) immediately after the intervention ends and at 12 months after randomisation to identify any potential differences in completion between those randomly assigned to exercise and those randomly assigned to control
4. Identify a primary outcome and estimate the sample size for a subsequent definitive trial
5. Refine a framework to facilitate conducting a cost-effectiveness analysis of the intervention
6. Conduct post-intervention interviews with participants to refine the design and delivery of the supervised exercise programme.

## Methods/Design

### Study design

The FISCU (Feasibility of Implementing Supervised exercise training alongside Compression therapy in people with venous Ulceration) study is a mixed methods feasibility study involving an exploratory randomised controlled trial with a nested qualitative component. Eighty adults who are receiving lower-limb compression for a new venous ulcer will be randomly assigned 1:1 to receive usual care (compression only) or usual care plus a 12-week supervised exercise programme. Participants will be followed up until 12 months after randomisation. The study is being run across two centres: Sheffield and Lincoln, UK. The study is registered with the International Standard Randomized Control Trial Number Register (ISRCTN10205425) while the study protocol conforms to the Spirit Checklist (Additional file 1).

This study started in May 2014 and is due to last until May 2017. The trial is sponsored by the Sheffield Health and Social Care NHS Foundation Trust, and ethical approval was granted by the NHS National Research Ethics Service, Yorkshire and the Humber (Sheffield) Committee (14/YH/0091), which co-ordinates ethical permissions across the following study and recruitment sites:

- Sheffield Hallam University
- Sheffield Health and Social Care NHS Foundation Trust
- Sheffield Teaching Hospitals NHS Foundation Trust
- Sheffield Clinical Commissioning Group
- University of Lincoln
- Lincolnshire Community Health Services NHS Trust.

### Recruitment of participants

In both Sheffield and Lincoln, participants will be recruited from community nursing and tissue viability teams or services, family doctor practices, community and outpatient leg ulcer clinics, and wound clinics. The primary mode of identifying potentially eligible patients will be through the weekly screening of primary care-based electronic record systems by practice nurses, tissue viability nurses, or specially trained administrators. Poster advertisements will also be used.

Eligible individuals will be sent an invitation letter, a brochure about the study, containing an invitation letter (including the contact details of a research team member), and a reply slip. For those recruited directly from primary care, the invitation letters are sent by the primary care practice where the search was conducted. For those recruited from existing databases, the invitation is sent from the chief investigator. Individuals who are interested in taking part are asked to return a reply slip directly to the FISCU research team. Patients who satisfy telephone pre-screening will be invited to the Centre for Sport and Exercise Science at Sheffield Hallam University or the Human Performance Centre at the University of Lincoln for their first formal study visit, during which they will be habituated with the trial, provide written informed consent (obtained by a research assistant or clinician), be confirmed for eligibility by a clinician, and complete baseline assessments.

### Eligibility criteria

Patients are eligible for the trial if they:

- are at least 18 years of age
- have at least one venous leg ulcer of primarily venous aetiology (determined by a clinician) with a maximum diameter of  at least 1 cm
- have an ankle brachial pressure index (ABPI) of at least 0.8 (recorded within the previous 3 months)
- are able and willing to tolerate lower-limb compression.

Patients are excluded from the trial if they:

- are unsuitable or unable to exercise (determined by a clinician)
- are unable or unwilling to tolerate lower-limb compression
- have insulin-controlled diabetes mellitus
- are pregnant
- have coexisting skin conditions, vasculitis, deep venous occlusion or malignant/atypical ulceration
- require major surgery
- have a leg ulcer with a maximum diameter of less than 1 cm
- have had an ulcer at the same site within the previous 3 months
- are unable or do not wish to consent to participation in the trial.

### Baseline measurements

In visit 1, after written informed consent has been obtained and eligibility confirmed (which will include a medical examination), the following baseline measurements will be recorded:

- demographic data, including age, sex, and socioeconomic status
- clinical history, including incident or recurrent ulcer, duration of ulcer disease, duration of current ulcer (oldest ulcer and reference ulcer if different), previous operations, comorbidities, current medications, height, weight, ankle and calf circumference
- ulcer size: measurement of ulcer size will involve taking a leg ulcer tracing by using a fine-nibbed indelible pen onto a conformable acetate film with a pre-printed grid. When a participant has multiple venous ulcers, all ulcers will be traced, and the eligible ulcer with the largest surface area will be deemed the reference ulcer for the trial. The reference ulcer and any other ulcers will be drawn onto a leg diagram. A digital image of the reference ulcer will also be taken.
- ABPI: a Doppler-determined measurement of ABPI will be performed according to the procedures of Aboyans et al. [17] unless a reading of less than 3 months old has been obtained from clinical records.
- baseline exercise history
- health status: EQ-5D-5L [18]
- disease-specific quality of life and symptoms: VEINES-QOL/Sym [19]
- lower-limb cutaneous microvascular function (see Outcome Measures section for detailed description) [14, 20]
- physical fitness, using three items from the Senior Fitness Test (see Outcome Measures section for detailed description) [21] and ankle range of motion assessed by using a bi-plane ankle goniometer (maximum dorsiflexion through to maximum plantarflexion).

### Randomisation and masking

After completion of baseline assessments, participants will be randomly assigned 1:1 to an intervention group or a control group. The research assistant will request (via email) a participant’s allocation from the trial statistician (AG) who manages the randomisation schedule. The statistician is not involved in the recruitment or data-gathering processes and is based in a different location to where the study visits are completed. Participants will be stratified by ulcer size (maximum ulcer diameter of either between 1 and 3 cm or greater than 3 cm in any direction) with permuted blocks of variable size, because this criterion is a known predictor of ulcer healing [22]. We could not mask participants or intervention supervisors to the allocated treatment. Outcome assessors will be blinded to group allocation.

### Withdrawals

Participants will be considered to be withdrawn from the trial if they request to leave the trial, if they are lost to follow-up, or if they die before completing the 12-month follow-up. Participants will be considered to be withdrawn from the allocated treatment if they no longer receive the supervised exercise training if allocated to the exercise group or if they no longer receive compression therapy regardless of which group they are in. Treating nurses will be asked to keep the participant with their original compression therapy (i.e., bandaging or stockings) unless there are objective clinical reasons for changing. Withdrawals from the trial and from the allocated treatment will be included in the analysis by intention-to-treat.

### Supervised exercise programme

A description of the supervised exercise programme is provided separately (Additional file 2). Participants allocated to the intervention group will be invited to attend three sessions of supervised exercise each week for 12 weeks (total of 36 sessions) at one of the two study exercise training facilities (Sheffield Hallam University and University of Lincoln). Sessions will typically be delivered on Mondays, Wednesdays and Fridays to allow sufficient recovery between sessions, although the weekly training structure will be flexible to work around participants’ commitments. The 12-week programme duration was chosen on the basis of a median ulcer healing time of 9 to 11 weeks for patients receiving compression therapy [23]. Sessions will be performed in the late morning, afternoon or early evening, given the higher occurrence of adverse cardiovascular events during early-morning exercise [24]. A maximum of 14 weeks will be allowed for the participants to complete the 36 sessions in case sessions are missed because of, for example, illness or holiday.

Each exercise session will last approximately 60 minutes and will comprise a combination of aerobic, resistance and flexibility exercises. Each session will begin and end with 5 minutes of low-intensity treadmill walking or cycling for a warm-up and cool-down, respectively. The aerobic component will last approximately 30 minutes, and whether the exercise mode is treadmill walking, cycling, or a combination of both is determined by the physical function and preference of participants. Treadmill hill-walking will be the preferred mode since it promotes greater recruitment of the calf musculature than cycling. The intensity will be moderate and this is due to evidence that moderate-intensity exercise training improves cardiorespiratory fitness, cardiovascular health, venous vascular function and skin microvascular function [14, 25, 26] and the fact that it will likely be better tolerated than high-intensity exercise. The intensity of exercise will be guided by the use of Borg’s 6- to 20-point exertion scale [27]; participants will exercise at an exertion level of 12 to 14 (somewhat hard), which equates to the ventilatory threshold [28]. The use of relative intensities will ensure that the exercise programme has inherent progression as the participants become fitter. Perceived exertion, heart rate (via telemetry) and exercise settings (e.g., treadmill speed and gradient) will be recorded at 5-minute intervals during aerobic training to allow accurate quantification of the exercise stimulus and progression of the programme over time.

Resistance and flexibility exercises will be performed for approximately 20 minutes in order to improve calf muscle pump function, leg (predominantly calf) muscle strength, and joint (predominantly ankle) mobility. Resistance exercises will predominantly involve dynamic body-weight exercises with or without the use of dumbbells and stability balls (e.g., calf raises and partial squats). Exercise will be performed for two or three sets of 10 to 15 repetitions to the point of moderate muscle fatigue [29]. For flexibility, static stretches will be performed for all of the major muscle groups of the legs, for a total of 60 seconds per muscle group (comprising 3 × 20-second stretches), held at the point of mild discomfort [29].

A trained exercise physiologist will supervise each session, and there will be a maximum of four participants per session to help ensure patient safety, successful delivery of the exercise session and collection of data. Compression garments (stockings/bandages) will be monitored during each exercise session: if affected by exercise, participants will be referred to the tissue-viability nursing team for re-application, and additional visits will be noted for the health-economics analyses. An experienced clinical exercise physiologist (GT) will be responsible for ensuring treatment fidelity of the exercise programme at both training sites.

After the completion of the intervention period, all exercise group participants will receive a personalised leaflet including an exercise programme that they could manage on their own. Adherence to those instructions will be monitored at follow-up visits.

### Usual care

Participants from both groups will receive standard compression therapy directed by experienced tissue-viability nurses, according to their individual circumstances and following standard local practice (e.g., multi-layered compression bandaging or two-layer compression stockings as well as standard advice to elevate the affected leg). Patients will be reviewed in clinics as often as is deemed clinically necessary.

### Study outcomes

All staff of the respective trial sites have been trained on the study protocol, informed consent and assessment data collection procedures. Study-specific standard operating procedures have also been developed to help ensure consistent data collection procedures between different outcome assessors and sites. Outcome assessors will be blinded to group allocation.

Follow-up visits for assessment of outcome measures will be completed 3 months (i.e., soon after the end of the supervised exercise programme) and 12 months after randomisation. A postal questionnaire follow-up will also be conducted 6 months after randomisation.

### Feasibility outcomes

The main focus of this study is acceptability and feasibility of procedures for recruitment, allocation, measurement and retention and for the intervention procedures. Recruitment rates will be measured as rate of invited participants who are eligible and consenting and will be reported in a CONSORT (Consolidated Standards of Reporting Trials) participant flowchart. Acceptability of allocation procedures will be assessed by examining reasons for dropout in discontinuing participants and comparing attrition rates between the two study groups. Suitability of measurement procedures will be evaluated on the basis of completion rates and reasons for missing data. Attrition rates will be established as discontinuation of intervention and loss to follow-up measurement for all conditions. The acceptability of the exercise programmes will be assessed by using session attendance and compliance data and participant feedback via one-to-one semi-structured interviews conducted with a sub-group of participants after the 3-month follow-up visit. The safety of exercise training will also be assessed by exploring reasons for dropout from the exercise programme and the number and type of adverse events that occur in each group.

### Secondary outcomes

#### Time to reference ulcer healed

Healing will be defined as complete epithelialisation in the absence of scab/eschar. The treating nurse will inform a study investigator when the reference ulcer and last ulcer (if applicable) have healed. A digital photograph of the reference/last ulcer site will be taken for validation purposes at healing and 7 days after healing. These images will be assessed ‘blind’ for confirmation of healing.

#### Proportion of patients healed

The proportion of patients healed will be assessed 3, 6 and 12 months after randomisation. This will allow direct comparison with other trials.

#### Percentage and absolute change in ulcer size

Percentage and absolute change in ulcer size will be measured 3 and 12 months after randomisation. The data collected will allow the determination of reduction in ulcer area in patients who do not achieve complete ulcer healing. Measurement of ulcer size will involve tracing on acetate films, as described above.

#### Proportion of time patients are ulcer-free

Reduction in recurrence would help reduce the prevalence of this condition and thus cost. Crude recurrence rates are potentially biased by any difference in healing rates associated with the two groups (exercise or control) since if one group has more rapid healing, then people in that group are at risk of earlier recurrence. To account for this, we will use the proportion of time that patients are ulcer-free as the clinically important measure since it is a function of both healing and recurrence and it is important for patients. Recurrence data will be collected 3, 6 and 12 months after randomisation.

#### Health-related quality of life

Health-related quality of life will be assessed 3, 6 and 12 months after randomisation by using the EQ-5D-5L and VEINES-QOL/Sym. The EQ-5D is a generic measure of health status, in which health is characterized on five dimensions (mobility, self care, ability to undertake usual activities, pain and anxiety/depression) [18]. Participants will be asked to describe their level of health on each dimension by using one of five levels: no problems, slight problems, moderate problems, severe problems or extreme problems. The EQ-5D has been validated in the UK and was used in the Venous leg Ulcer Study III (VenUS III) and VenUS IV trials [5, 30]. The VEINES-QOL/Sym is a disease-specific quality-of-life instrument for use in venous diseases of the leg [19].

#### Lower-limb cutaneous microvascular function

Laser Doppler fluximetry and iontophoresis will be used to assess cutaneous microvascular function [31] in the gaiter area of the affected leg. We will assess areas of skin that are in close proximity to the ulcer but at least 3 cm away from the edge of the ulcer. Assessments will be performed in a temperature-controlled room (22–24 °C) with the participants in a supine position and the leg elevated at 30°. Compression garments will be removed only if patient care is not compromised (e.g., removable compression stockings are worn). Heart rate (Sports Tester; Polar, Kempele, Finland) and blood pressure (left arm; Dinamap Dash 2500; GE Healthcare, Wauwatosa, WI, USA) will be monitored at 5-minute intervals throughout the protocol. The two drug delivery electrodes (PF383; Perimed AB, Jarfalla, Sweden) will be positioned over healthy-looking skin, approximately 4 cm apart; one will contain 100 μl of 1 % acetylcholine chloride (ACh) (Miochol-E; Novartis, Stein, Switzerland) and the other 80 μl of 1 % sodium nitroprusside (SNP) (Nitroprussiat; Rottapharm, Monza, Italy), and de-ionised water will be used as the solvent. A battery-powered iontophoresis controller (PeriIont PF382b; Perimed AB) will be used to provide the charge needed for the simultaneous delivery of ACh and SNP. A 4-minute stable recording of baseline flux will be followed by administration of the two agents in accordance with the following protocol: 0.2 mA for 10 seconds (i.e., 2 mC), 0.2 mA for 15 seconds (i.e., 3 mC), 0.2 mA for 20 seconds (i.e., 4 mC), and 0.3 mA for 20 seconds (i.e., 6 mC), with 4-minute intervals between each dose. To obtain an index of skin blood flow, cutaneous red cell flux will be measured by placing an iontophoresis laser-Doppler probe (PF481-1; Perimed AB) connected to a laser Doppler fluxmeter (PF5001; Perimed AB). Responses to ACh and SNP will be used as indicators of the changes occurring in the endothelial-dependent and -independent vasodilatory function, respectively. The process will be repeated in the gaiter area of the non-affected leg following a 10-minute resting period.

#### Physical fitness – Senior Fitness Test

The Senior Fitness Test is a widely used test battery for evaluating the effects of exercise interventions in older adults and consists of six sub-tests which measure the physical abilities needed to perform activities of daily living [21]. However, three of the sub-tests—for upper-body flexibility, upper-body strength, and agility—will not be used, as these aspects of physical fitness are not targeted within the exercise intervention. Therefore, the tests used in this trial will be (i) 30-second chair-stand test for lower-body muscular strength and endurance (the number of times in 30 seconds a participant can stand fully from a seated position without using their arms), (ii) chair sit and reach test for lower body flexibility (from a sitting position at front of chair, with leg extended and hands reaching toward toes, the number of centimetres (+ or −) between extended fingers and tip of toe), and (iii) the 6-minute walk test for aerobic fitness (the maximum distance a participant can walk in 6 minutes). All tests will be measured once, except the sit and reach tests, which will consist of a practice, then two trials. The Senior Fitness Test has acceptable test-retest reliability (R = 0.81–0.98) and construct validity against a range of indicators, such as age and exercise status, and criterion validity (r = 0.71–0.82) [32].

#### Adherence to background clinical therapies

All participants will be asked to complete specially developed, self-reported diaries for their adherence to leg elevation instructions, usage of compression therapy and contact time with the tissue-viability nursing team to assist the full-study and health-economic analysis. This procedure will be supported by weekly phone calls from a research team-member at all stages.

### Health economics

The broader aim is to undertake a comprehensive cost-utility analysis with both NHS and societal perspectives. However, in this feasibility study, the primary objective is to account for as well as make fair assessment and valuation of costs (direct and indirect costs) and benefits (quality-adjusted life-year (QALY) gains) as a result of the 12-week exercise intervention. The major cost drivers will be patient’s use of NHS and private health services, the patient’s time and their travel costs and intervention delivery costs.

#### Cost of intervention

This will include staff time, including exercise instructor related to intervention delivery, resources and equipment used and room costs as well as any incentive given to patients to adhere to the exercise session.

#### Costs to patients in the intervention group

This will be based on the distance to the exercise training facility and travel costs, time taken off from work to attend the exercise session and any additional equipment/aids/shoes and bandage/dressing purchased to undertake exercise sessions.

#### Difference in resource use between intervention and control groups

The resource use questionnaire will be completed by intervention and control groups at 3-, 6- and 12-month intervals to elicit information on utilisation of NHS and private health-care services related to leg ulcer treatment. It collects information on the number of visits, travel time and cost, and cost to the patient for the following uses: General practitioner; Doctor at leg ulcer clinic; NHS Hospital consultant/specialist; Doctor/nurse at accident and emergency; NHS Physiotherapist; Private Physiotherapist; Private doctor/consultant; Private chiropractor; Psychologist/counsellor; and Any other. Similarly, the number of times and the costs to the patient for undertaking diagnostic tests (e.g., x-rays, computed tomography scan, magnetic resonance imaging scan, blood test and any other tests) and for medications (e.g., pain killers, sleeping pills and antibiotics) and other aids (e.g., compression hosiery or bandages) related to leg ulcer will be collected. Furthermore, number of days of hospitalisation related to leg ulcer and data on costs for any equipment purchased for speedy recovery from leg ulcer will be collected.

#### Difference in time taken off from work between intervention and control groups

The patient’s work status, employment type (full-time, part-time, self-employed, unpaid work, unemployed and inactive) and the number of days off from work because of leg ulcer will be accounted for in presenting the societal perspective on costs and benefits of the intervention. The changes in the employment status because of leg ulcer will be recorded during the intervention and follow-up period.

#### Differences in quality of life between intervention and control groups

The new EQ-5D-5L questionnaire will be used to measure quality of life at baseline and at 3-, 6- and 12-month intervals. Using the standardised general population-based tariff, the scores will be converted into QALYs and then compared between intervention and control groups.

#### Incremental cost-effectiveness ratio

To measure the net effect of intervention, the incremental cost-effectiveness ratio will be derived from relating differences in costs to benefits between intervention and control groups during a 12-month period. We will use standard published sources for unit costs where available and supplement this with local sources if required. We will use the same methods of analysis as in a definitive trial, including imputation methods for missing data, analysis of uncertainty and non-normal distributions: that is, standard statistical analysis of costs will be performed to test for statistical significance of results, while bootstrapping would be performed to estimate the confidence interval (CI) of average interventional costs.

### Qualitative component

The aim of the qualitative component is to explore participants’ experiences of having a venous ulcer and the acceptability of the exercise intervention and study procedures. A sub-sample of 16 participants will be recruited by using purposive sampling (mixture of genders, younger and older participants from intervention and control groups). Semi-structured interviews of up to 1 hour will be conducted with participants either face-to-face or via telephone (participants’ preference) after the 3-month follow-up visit. The following topics will be explored:

- participants experiences’ of leg ulceration
- experiences of treatment and advice given
- participants’ preference for trial allocation
- participant acceptability of the exercise intervention and study procedures.

Transcripts will be analysed thematically by using framework analysis as this approach provides a systematic process to analyse qualitative data. The in-depth interview offers an opportunity to investigate in detail participant’s personal perspectives of their leg ulcer experiences set within their personal frame of reference. The interview method also means that there will be time to explore participant’s motivations, attitudes, intervention impact and experiences in greater detail than that afforded by a focus group setting [33]. Framework analysis [34] offers an approach that is somewhat inductive; that is, it is based in the original interview transcripts but framed within the “constraints” of an *a priori* framework. An audio recording of the interviews will be made and then transcribed verbatim, and the five stages of framework analysis (familiarisation, identifying a thematic framework, indexing, charting and mapping) will be followed.

### Statistical analysis

The sample size for a feasibility study should be adequate to estimate critical metrics, needed to assess the feasibility of conducting the definitive study, with sufficient precision. Herein, the critical metrics are the consent rate (i.e., the proportion of eligible patients who consent to participate and be randomly assigned), ulcer-healing rates and compliance with treatment, and attrition. Forty patients in each group will provide a sufficiently precise (within 15 percentage points for a 90 % CI) estimate of the proportion willing to be randomly assigned, assuming 35–40 % intention to be randomly assigned. A sample size for an adequately powered randomised controlled trial will be determined from the results of this trial.

Descriptive statistics will be used to characterise the groups at baseline and to present the feasibility outcomes described above. Although determining differences in clinical outcomes between the two arms is not the primary purpose of this trial, comparisons will be undertaken to investigate the feasibility of studying these outcomes and to calculate estimates for the likely effect sizes and 95 % CIs. Analyses will be conducted in SPSS by using the principles of intention-to-treat. Significance tests will be two-sided at the 5 % level. Healing rates will be compared by using the Kaplan-Meier method. Hazard ratios and 95 % CIs will be presented for ulcer healing time by using Cox regression analysis models. For other outcomes, the intervention and control groups will be compared by using linear or logistic regression (depending on the type of outcome) adjusting for ulcer size and any other important baseline covariate. Relevant assumptions will be checked for all models. For the potential primary outcomes for a definitive trial, the likely effect of the exercise intervention will be estimated via standard intervention minus control comparisons, accounting for the type of variable and its distributional properties. We will examine whether the CIs include potentially clinically important effects in outcomes between groups. As this is a feasibility study, these comparisons are exploratory and are intended to inform the subsequent, definitive trial.

### Criteria for success

This feasibility trial will be deemed successful and lead to the development of a proposal for an adequately powered randomised controlled trial if:

(i) An appropriate primary outcome variable is defined
(ii) At least 67 % of randomly assigned patients in the exercise group are compliant with the intervention (defined as at least 75 % of the scheduled sessions are completed as planned)
(iii) Loss to follow-up at 12 months is less than 20 %
(iv) Patient preferences are not so strong that they result in the conclusion that a randomised controlled trial is not a feasible design.

The criteria for success will provide the basis for interpreting the results of this study and determine whether it is appropriate to proceed to a full trial and (if appropriate) what modifications need to occur for a full trial to be feasible.

### Project management

The management of this study will be the responsibility of the research management group, consisting of the chief investigator (MK), all co-applicants (including GT, HC, JM and AG) and all research staff. Operational management will be the responsibility of the research team consisting of the chief investigator, the principal investigator in Lincoln (GM) and research staff, meeting once a week to ensure adherence to planned timescale, adherence to the intervention and detailed plans for data management and analysis. A trial steering committee consisting of independent clinical (Ian Chetter and Brenda King) and academic members (Jude Watson and Catherine Hewitt) and the chief investigator (MK) will also meet at least twice a year. This committee is responsible for the overall management and oversight of the trial. Because of the low-risk nature of this trial (both treatments being assessed were already being used routinely in clinical practice), we did not judge it necessary to have a separate Data Monitoring and Ethics Committee to oversee the trial. This study was co-adopted to the Primary Care and Dermatology specialities of the UK Clinical Research Network Portfolio (UKCRN ID 16665), which provides research infrastructure resources, including Research Nurse support for recruitment.

## Discussion

Venous leg ulcers may take many months to heal (and some do not) and during this time they result in significant suffering for patients and represent substantial cost to the NHS. At present, compression is the main treatment for venous ulceration and few additional therapies exist to improve healing. The FISCU study will provide preliminary evidence about the effectiveness of supervised exercise training as an adjunct therapy for enhancing ulcer healing, physical fitness and health-related quality of life in people with venous ulcers. The findings will inform a future definitive multi-centre randomised controlled trial with a larger sample size. Strengths of the current preliminary study include follow-up through 12 months after randomisation, use of a strategy to assess the fidelity of the intervention, and inclusion of relevant objective and patient-reported outcome measures. A limitation of this study is that there are only two recruiting sites and this may limit the generalisability of the findings. The external validity of university-based exercise training may also be questionable given that supervised exercise training is unlikely to be delivered in this setting if it were to become an established part of the management of venous ulceration. Benefits of using a university setting in the current study include the availability of dedicated exercise facilities for research purposes and free parking. These issues of generalisability will be addressed in the future main study by having more (probably more than 10) trial sites and using existing NHS resources for delivering the intervention (e.g., hospital or community-based cardiac rehabilitation programmes).

## Trial status

Recruitment started in July 2014 and is ongoing.

## Additional files

Additional file 1:
**SPIRIT 2013 Checklist.** Information about how FISCU conforms to the SPIRIT 2013 checklist. *FISCU* Feasibility of Implementing Supervised exercise training alongside Compression therapy in people with venous Ulceration. (PDF 39 kb) Additional file 2:
**Details of the implemented supervised exercise programme.** A detailed account of the implemented supervised exercise programme. (PDF 33 kb)

## Abbreviations

ABPI: Ankle Brachial Pressure Index
ACh: Acetylcholine chloride
CI: Confidence intervals
CVI: Chronic venous insufficiency
EQ-5D-5L: EuroQol 5-dimension (5L) health status questionnaire
FISCU: Feasibility of Implementing Supervised exercise training alongside Compression therapy in people with venous Ulceration
NHS: National Health Service
QALY: Quality-adjusted life-year
SNP: Sodium nitroprusside
VEINES-QOL/Sym: VEnous INsufficiency Epidemiological and Economic Study-Quality of life and symptoms questionnaire
VenUS: Venous leg Ulcer Study
