# Supplementary material for: Supervised exercise training as an adjunctive therapy for venous leg ulcers: study protocol for a randomised controlled trial
Source: Trials. 2015 Oct 6;16:443. doi: 10.1186/s13063-015-0963-z (PMC4595245; doi:10.1186/s13063-015-0963-z)
Supplement: Additional file 2: — Details of the implemented supervised exercise programme. A detailed account of the implemented supervised exercise programme. (PDF 33 kb) [file 13063_2015_963_MOESM2_ESM.pdf]

Table 1 Details of the supervised exercise programme

	**Details**
**Goals of the intervention**	The primary goal of the supervised exercise programme is to reduce ulcer healing time via training-induced improvements in calf muscle pump function, ankle range of motion, and lower-limb cutaneous microvascular function. Secondary goals are reduce cardiovascular disease risk, to improve health-related quality of life, and to improve several aspects of physical fitness including cardiorespiratory fitness, muscle strength and endurance, and flexibility.
**Materials used in intervention delivery**	Two motorized treadmills (Life Fitness 95T Achieve); two upright exercise bikes (Life Fitness 95C Achieve); pairs of dumbbells ranging 2-20 kg; one height-adjustable step; one medium-sized stability (Swiss) ball; one seated leg press machine (Life Fitness Signature Series); Theraband; Polar heart rate monitor; Borg 6-20 rating of perceived exertion scale; radio for music; water-dispensing machine; two large fans on stands
**Procedures used in the intervention**	Participants will be invited to attend 3 sessions of supervised exercise each week for 12 weeks (total of 36 sessions). Each exercise session will last approximately 60 minutes and will comprise a combination of aerobic, resistance and flexibility exercises. Sessions will typically be delivered on Mondays, Wednesdays and Fridays to allow sufficient recovery between sessions, and will be performed either in the late morning, afternoon or early evening. A maximum of 14 weeks will be allowed for the participants to complete the 36 sessions, for if sessions are missed due to, for example, illness or holiday.<br><br>Warm-up: Each session will begin with a 5-minute warm-up of low-intensity treadmill walking or cycling, determined by participants' physical function and preferences. The target for the warm-up period is for the participant to exercise at an exertion level of no higher than 11 (light) on Borg's 6-20 rating of perceived exertion (RPE) scale.<br><br>Aerobic component: The aerobic component will last approximately 30 minutes, with the exercise mode being treadmill walking, cycling, or a combination of both, determined by the physical function and preference of participants. Treadmill hill-walking will be the preferred mode, since it promotes greater recruitment of the calf musculature than cycling. The intensity of exercise will be guided by the use of Borg's 6-20 scale, with participants encouraged to exercise at an exertion level of 12-14 (somewhat hard).<br><br>Resistance component: Resistance exercises will be performed for approximately 15 minutes. Four exercises should be completed in each session: two targeting the calf muscles and two targeting the muscles of the thigh and hips. The exercises will predominantly involve dynamic body-weight exercises with or without the use of dumbbells and stability balls. An example calf exercise is the standing calf raise. Example thigh/hip exercises include partial squats and the chair sit-to-stand exercise. Exercises will be performed for 2-3 sets of 10-15 repetitions to the point of moderate muscle fatigue.<br><br>Cool-down and flexibility component: The participant should complete a 5-minute cool-down of low-intensity treadmill walking or cycling, as in the warm-up. Static stretches will then be performed for calves, quadriceps and hamstrings, for a total of 60 seconds per muscle group (comprising 3 × 20-second stretches), held at the point of mild discomfort.
**Intervention provider**	The exercise programme will be supervised by exercise physiologists who have several years' experience of exercise supervision and prescription. The exercise physiologists all received at least one day of training specific to the trial from one of the co-applicants (GT) who has completed trials of supervised exercise training in several different patient populations.

**Mode of delivery**	All sessions were supervised. A maximum of 4 participants to 1 supervisor is allowed, to ensure patient safety, successful delivery of the exercise session and all relevant data collection.
**Where the intervention is delivered**	The exercise programme is delivered at the Centre for Sport and Exercise Science at Sheffield Hallam University and the Human Performance Centre at the University of Lincoln. These centres have free parking and exercise facilities that are dedicated for research (i.e. they are not used by the public).
**How the intervention is personalised and adapted**	At the start of the programme, the exercise physiologist will work to determine which exercises the participants prefers and which are the most appropriate based on the participant's current levels of physical fitness and mobility. The initial goal is for participants to build up to completing three, one-hour sessions of exercise each week as described above. Once the session frequency and duration targets are being met, the intensity of the specific exercises will be progressed on an individual basis. For the aerobic component, the speed and incline of the treadmill and the resistance level of the bike will be increased to keep the participant within the RPE range of 12-14. For the resistance component, the weight or type of exercise used will be adapted so that the participant continues to reach the point of moderate muscle fatigue within 10-15 repetitions.
**Strategies to maintain intervention fidelity**	The timing of the sessions will be flexible to help participants achieve three sessions per week. Free parking is available at each site and participants receive up to £5 per visit towards travel expenses. Each session must be supervised by an investigator who has received specific training for this trial. One of the co-applicants (GT) will provide oversight to the delivery of the exercise intervention, and this will include regularly observing sessions and checking CRFs for errors.
**Processes for evaluating intervention attendance and compliance**	A specially-designed Exercise Session Case Report Form (CRF) will be completed every session. The following information will be written at the top of each of these forms: participant initials and trial ID number; date and time of session; session and week numbers; supervisor name(s). The supervisor will also record the type of compression garment being worn by the participant, or "none" if that is the case (note: the participant is allowed to complete an exercise session without compression garments being worn; however, they should be encouraged to wear them in future sessions). The participant will be fitted with a Polar heart rate monitor at the start of each session.<br><br>During the warm-up, aerobic and cool-down sections, RPE (Borg 6-20 scale), heart rate (via telemetry) and exercise type and settings (e.g. treadmill speed and gradient) will be recorded for the last 15 seconds of each 5-minute period. This will allow accurate quantification of the exercise stimulus and progression of the programme over time. Similarly, the types of resistance and flexibility exercises performed will be recorded, along with details of number of sets, repetitions and end-exercise RPE.<br><br>Compression garments (stockings/bandages) will be monitored during each exercise session: if affected by exercise, participants will be referred to the tissue-viability nursing team for re-application, with additional visits being noted for the health-economics analyses. If sessions are missed, the reason(s) for this will be documented on the CRF.
